# Autonomic vulnerability to biased perception of social inclusion in borderline personality disorder

**DOI:** 10.1186/s40479-021-00169-3

**Published:** 2021-11-18

**Authors:** Gerra Maria Lidia, Ardizzi Martina, Martorana Silvia, Leoni Veronica, Riva Paolo, Preti Emanuele, Marino Barbara Francesca Marta, Ossola Paolo, Marchesi Carlo, Gallese Vittorio, De Panfilis Chiara

**Affiliations:** 1 Department of Mental Health, AUSL of Parma, Parma, Italy; 2 Department of Medicine and Surgery, University of Parma, Parma, Italy; 3 Department of Psychology, University of Milano-Bicocca, Milan, Italy

**Keywords:** Respiratory sinus arrhythmia, Rejection bias, *Cyberball* paradigm, Polyvagal theory

## Abstract

**Background:**

Individuals with Borderline Personality Disorder (BPD) feel rejected even when socially included. The pathophysiological mechanisms of this rejection bias are still unknown. Using the *Cyberball* paradigm, we investigated whether patients with BPD, display altered physiological responses to social inclusion and ostracism, as assessed by changes in Respiratory Sinus Arrhythmia (RSA).

**Methods:**

The sample comprised 30 patients with BPD, 30 with remitted Major Depressive Disorder (rMDD) and 30 Healthy Controls (HC). Self-report ratings of threats toward one’s fundamental need to belong and RSA reactivity were measured immediately after each *Cyberball* condition.

**Results:**

Participants with BPD showed lower RSA at rest than HC. Only patients with BPD, reported higher threats to fundamental needs and exhibited a further decline in RSA after the Inclusion condition.

**Conclusions:**

Individuals with BPD experience a biased appraisal of social inclusion both at the subjective and physiological level, showing higher feelings of ostracism and a breakdown of autonomic regulation to including social scenarios.

## Background

Borderline Personality Disorder (BPD) is a severe mental illness affecting approximately 1% of the general population [1]. Social dysfunction represents one of the most enduring and challenging to treat feature of the disorder, which is not substantially affected by a symptomatic decrease or even remission over time [2]. In BPD, social impairment is fostered by a unique interpersonal hypersensitivity pattern, encompassing extensive preoccupation with real or imagined abandonment and rejection, and related distrustful perceptions of others as bad, malevolent, and excluding [3–5]. Therefore, clarifying the potential mechanisms fostering this peculiar way of processing interpersonal cues is a primary clinical and research goal in BPD study.

### Rejection bias in BPD

Recent studies evaluated BPD patients’ responses to varying degrees of interpersonal inclusion using *Cyberball*, a virtual ball-tossing game where participants can be socially excluded, included, or even over-included by others [6, 7]. Results indicate that patients with BPD do not merely “over-react” to actual social exclusion; rather, they feel rejected and experience greater rejection-related negative emotions than controls following objective interpersonal inclusion [8–10]. Moreover, individuals with BPD feel disconnected from others even when they face a condition of extreme interpersonal inclusion [11, 12]. Overall, these findings suggest a “misperception of social participation”: patients with BPD show biased processing of social inclusion, which makes them perceive rejection even in interpersonal situations that are objectively including.

### Autonomic correlates of rejection bias in BPD

To date, most BPD studies focused on subjective (i.e., explicit, self-reported) emotional reactions to the *Cyberball* experiment, while the underlying implicit pathophysiological mechanisms of this rejection bias are yet to be fully elucidated.

The Polyvagal Theory [13] provides a theoretical framework to study the Autonomic Nervous System (ANS) reactivity to perceived threatening interpersonal cues. According to such theory, the myelinated vagal system evolved to support flexible adaptation to environmental stimuli. When the environment is appraised as safe, at the cardiac level, the “vagal brake” increases the parasympathethic activity on the hearth, slows down the heart rate, and inhibits the more primitive ANS systems (i.e., the sympathetic nervous system and the unmielynated vagus) that promote fight/fly or freeze defense strategies. Ultimately, this serves to support effective social engagement behaviors. In this way, prosocial-affiliative interactions can adaptively emerge and persist over time in safe contexts.

The dynamic functional impact of the myelinated vagal fibers on the heart is reflected by the amplitude of the Respiratory Sinus Arrhythmia (RSA), a naturally occurring rhythm in the cardiac cycle at approximately the frequency of spontaneous breathing [14–16]. Thus, measurement of the amplitude of RSA provides an assessment of the state of the vagal brake: increased vagal influence on the heart corresponds to high or increased RSA. By contrast, in challenging or threatening situations, the vagal brake is withdrawn, leading to physiological states that support the fight, flight, or freeze behaviors but inhibiting social engagement behaviors. This vagal withdrawal is reflected in RSA decreases. Thus, high RSA at rest and in safe environments and the appropriate RSA suppression in the face of real environmental risks represent a marker of successful self-regulation. Notably, this flexible and adaptive increase or decrease in RSA crucially depends on the environmental risks’ accurate appraisal.

The neural ability to distinguish environmental features that are safe, dangerous, or life-threatening is called neuroception, a process of neural detection of risk that does not require conscious awareness [13]. When neuroception is impaired, the ANS fails to distinguish between safe and dangerous contexts accurately: thus, the environment may be appraised as dangerous when it is safe. This leads to a mismatch between the actual risk of the environment and the neurophysiological state, resulting in an inability to appropriately inhibit the defense systems and maintain prosocial behaviors in safe environments. It is possible to measure such mismatch by assessing RSA both at rest and during various environmental challenges: low RSA in the absence of environmental demands or in response to stimuli that are not threatening would suggest that individuals physiologically appraise and react to safe environments *as if* they were actually unsafe [13].

In this regard, accumulating evidence indicates that patients with BPD exhibit low cardiac vagal tone at rest [17–21], indicating that they present a constant physiological condition of preparedness to face threats and danger. In the same vein, having low RSA at rest mediates the association between BPD symptoms and reactive aggression in a non-clinical population [22], suggesting that impaired vagal control leads to maladaptive social behaviors in individuals with BPD features.

Three other studies on BPD examined RSA reactivity, that is, RSA change in response to various experimental stimuli, like film clips of varying emotional content [23], mental arithmetic tasks [18], and standardized film and idiographic imagery paradigms [17]. Overall, these studies found that, among participants with BPD, RSA remained as low as at baseline [17] or even decreased during the experiment [18, 23]. These studies suggest that engaging in an emotional or cognitive experimental task induces, among patients with BPD, a physiological state that promotes defensive behaviors, with phylogenetically older ‘fight-or-flight’ response, rather than a visceral state that supports self-regulation and spontaneous social engagement behaviors.

To our knowledge, no study yet evaluated RSA reactivity in response to varying degrees of social inclusion in BPD. Such inquiry could clarify whether BPD patients subjectively perceive including social scenarios as if they were rejecting by reacting to them with a breakdown of the self-regulation and socialization capacities, rooted in the myelinated vagal system’s activity.

It is also important to examine whether such hypothesized in including situations, associated with vagal withdrawal, truly represents a BPD-specific alteration, by comparing BPD with a clinical and medicated control group, with similar illness duration. In this regard, patients with Major Depressive Disorder (MDD) have also been found to exhibit peculiar responses to the *Cyberball* experiment and altered RSA patterns as compared with Healthy Controls (HC).

As compared with HC, patients with active MDD (i.e., during full-blown depressive episodes) experience a greater sense of threat to psychological fundamental needs after social exclusion [24–29]. Interestingly, although one study also found that MDD patients report greater perception of threat after social inclusion than HC [30], another study argued that such rejection bias in including situations among patients with active MDD was explained by BPD comorbidity [31]. Only one study evaluated how patients with MDD in the remission phase (rMDD) react to the *Cyberball* experiment [32]. However, this study assessed social distress related to the task as a whole, and not after the inclusion and ostracism conditions. Thus, it is not known yet whether the increased sensitivity to ostracism (and possibly the rejection bias) showed by MDD patients during an active depressive episode would persist even in the euthymic phase, thus representing a trait-based phenomenon rather than just a state-dependent phenomenon that is apparent only during full-blown episodes.

With respect to RSA findings, patients with active MDD were found to exhibit low resting RSA and atypical RSA reactivity to various laboratory stressors. Further, during the remission phase patients with MDD exhibit low resting RSA but not altered RSA reactivity to laboratory tasks [33–38]. Thus, low RSA at rest seems to represent a stable, trait-like feature of MDD, which persists even during the euthymic phase, when patients with rMDD may still exhibit distinctive clinical features, such as sub-threshold psychopathology [35], peculiar personality styles [36], or symptomatological scars of previous episodes [37]. Conversely, altered RSA reactivity during active phases of depression is likely to represent a state-effect of full-blown depressive psychopathology [34]. However, no study yet measured RSA reactivity to *Cyberball* in MDD.

Thus, it is not yet clear whether MDD patients exhibit peculiar patterns of responses to *Cyberball* associated with altered RSA reactivity, which persist at the remission of depressive episodes.

Therefore, in this study we compared BPD patients, with no current depressive episode, with rMDD patients on maintenance treatment, with no BPD comorbidity, to investigate whether RSA alterations following the *Cyberball* conditions could represent a stable, trait-like element that could distinguish the clinical groups, over and above the confounding effect of full-blown depressive symptomatology. Importantly, such comparison also allows for controlling for the potential confounding effect of sub-threshold depressive symptoms. Patients with BPD often present with depressive symptoms, although known to be transient, stress reactive and arising from a primary diagnosis of BPD [39, 40]; in the same vein, patients with rMDD also may experience inter-episodic depressive psychopathology [35].

### The present study

This study investigated whether patients with BPD, compared to HC and patients with rMDD, show an altered emotional response associated with an altered vagal reactivity after Cyberball conditions of Social Inclusion and Ostracism, as well as 10 min after Ostracism (Reflective stage). Based on previous research, three main predictions guided our investigation.

First, we expected to replicate the finding that BPD patients, compared to healthy and clinical controls, would report reduced levels of satisfaction of fundamental psychological needs (e.g., the need to belong) even in including situation both immediately after Ostracism, as well as at the Reflective stage. This would confirm a biased perception of social inclusion at the subjective (i.e., explicit) level in BPD.

Second, we expected that patients with BPD, compared to healthy and clinical controls, would exhibit reduced RSA at rest (i.e., before starting the game), indicating stable difficulties in social predisposition at the physiological level. Moreover, we expected that at the ANS level, patients with BPD would show a further decrease in RSA after the Cyberball Inclusion condition than baseline RSA. This would indicate that individuals with BPD physiologically respond to including social situations *as if* they were threatening, with a dysfunctional withdrawal of the vagal brake that leads to increased physiological arousal, mobilizing defensive reactions but impeding successful social engagement.

Finally, we hypothesized that a higher perception of threat to fundamental psychological needs induced by the *Cyberball* task would be associated with higher physiological arousal as indicated by vagal withdrawal (i.e., a more substantial decline in RSA).

## Methods

### Participants

This study involved 30 patients with BPD, 30 patients with rMDD, and 30 HC. Patients were recruited at the psychiatry outpatient services of Parma Local Health Agency (Parma, Italy) from January 2016 to September 2018. HC, matched for age and gender with patients with BPD, were recruited through advertisements in meeting places in the local community.

### Inclusion and exclusion criteria

Inclusion criteria were: 1) age 18–65 years; 2) native Italian speaker or proficient in Italian; 3) for the clinical groups, meeting the diagnostic criteria for rMDD or BPD, assessed by the Structured Clinical Interview for DSM-5 disorders, Clinician Version (SCID-5-CV) [41] and the Structured Clinical Interview for DSM-5 Personality Disorders (SCID-5-PD) [42], respectively; 4) scoring< 7 on the 21-item Hamilton Rating Scale for Depression (HAM-D) [43] and < 7 in the Hamilton Anxiety Rating Scale (HAM-A) [44].

Exclusion criteria were: 1) any cardio-respiratory diseases and treatment that directly affect the ANS’s function (e.g., sympatho-mimetic and para-sympatho-mimetic drugs, alpha and beta receptors blockers, and anti-muscarinic drugs); 2) cognitive impairment, assessed by the Wechsler Adult Intelligence Scale Matrix Reasoning Subtest [45]; 3) any current diagnoses of Schizophrenia spectrum and other Psychotic Disorders, Bipolar Disorders, Anxiety Disorders, Post-Traumatic Stress Disorder, Somatic Symptom and related Disorders and Eating Disorders. For the BPD group, both current and lifetime MDD comorbidity also was an exclusion criterion. However, for both clinical groups, we included patients with other previous lifetime disorders, though fully remitted at the study time (i.e., Adjustment disorders, Substance-related disorders, Eating Disorders, Obsessive-Compulsive Disorder). Patients were not excluded for regular psychotropic medication use.

Participants were told that the researchers were investigating “Mental visualization and individual differences in heart rate and psychological responses.” This cover story is thought to maximize the experiment’s ecological validity [46]. Participants gave written informed consent to participation and, after completion of the experiment, were extensively debriefed and given detailed information about the study and its purposes, with the opportunity to have their data deleted should they wish so.

The total sample size collected (*N =* 90) exceeded the minimum amount required (*N* = 54) estimated using a priori sample size calculation, obtained for repeated-measures analyses of variance (ANOVA) considering both within and between interactions (1-ß = .95, α = .05, effect size F = .25). The sample size was computed with G*power [47] based on the effect size of previous studies that compared BPD with two other clinical groups [48]. We enlarged the a-priori required sample size up to 30 per group to account for covariates, such as age, Body Mass Index (BMI), alcohol and tobacco consumption, known to affect RSA.

### Psychometric assessment

All participants completed a general demographic questionnaire on age, gender, BMI, physical activity, educational level, occupational and marital status, and habitual consumption of psychotropic substances (alcohol, caffeine, and nicotine).

Psychosocial functioning was assessed with the Global Assessment of Functioning Scale (GAF) [49].

### Experimental procedure

Participants were led into a quiet and soft illuminated room and were instructed to relax and remain seated comfortably. At the beginning of the experimental session, participants were instructed to sit quietly with their eyes open, and a 2-min resting baseline electrocardiogram (ECG) was recorded to assess RSA at rest.

**Fig. 1:**
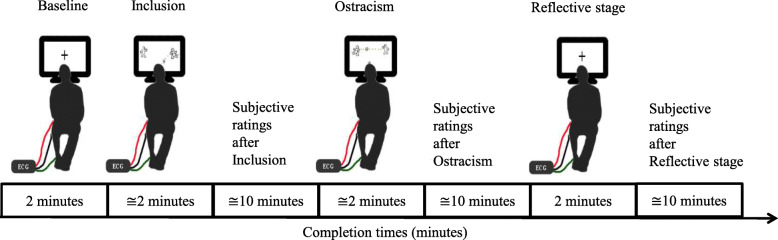
Experimental procedure

Subsequently, they participated in a *Cyberball* experiment and completed different measures of their current emotional state. ECG recordings were collected over the entire duration of the experimental session to extract phasic autonomic measures (i.e., RSA reactivity). Please refer to Fig. 1 for a graphical display of the experimental procedure.

### Cyberball experiment

Inclusionary status was manipulated using a classic paradigm called *Cyberball* (Cyberball (version 4.0) [Software] available from https://cyberball.wikispaces.com). This virtual ball-tossing game has been developed to induce feelings of ostracism in controlled settings [7]. Following the typical procedure [46], participants were told that investigators were interested in the effects of mental visualization on a subsequent task and that a good way to warm up was to engage in a mental visualization exercise with other online players. In actuality, these two other players were not real; instead, they were computer-controlled confederate players, identified using a name. All participants were enrolled in two consecutive conditions of the *Cyberball* game: initially, they were included in a belonging game (i.e., receiving the ball about a third of the time, roughly 33% of the total throws) and then ostracized (i.e., receiving the ball once from each computer-controlled player and then never again, roughly 10% of the total throws). The order of the Inclusion-Ostracism conditions was kept fixed for all participants (for a similar procedure, see: [50]).

### Manipulation checks

After each *Cyberball* condition, participants rated the percentage of throws (0–100%) they received during the game as a manipulation check. They were then asked to report how excluded (“I felt excluded”) and ignored (“I felt ignored”) they felt during each *Cyberball* session. Responses were rated on 10-point scales (ranging from 1 = not at all to 10 = very much). The two items were combined in an overall index of feelings of being excluded and ignored. Higher scores indicate greater feelings of ostracism.

### Subjective responses to the Cyberball game

Participants were asked to report their feelings three times: after *Cyberball* Inclusion, immediately after *Cyberball* Ostracism, and 10 min after completing the experiment (i.e., Reflective stage).

The Need-Threat Scale (NTS) measures satisfaction with the four fundamental needs potentially affected by ostracism in a 12-item scale: the need to have pleasant interactions with others (belonging), the need to believe others view us as worthy (self-esteem), the need to influence our social environment (control), and the need to avoid our fear of death by making an impact on the world (meaningful existence) [51]. Lower scores reflect the “ostracism distress”, e.g. a greater perceptions of threat to these fundamental needs. In this sample, the internal consistency of NTS was good across all assessment times (α_inclusion_ = .79; α_ostracism_ = .81; α_reflective stage_ = .87).

### Autonomic responses to the Cyberball game

Patients were fitted with three 10 mm Ag/AgCl pre-gelled adhesive electrodes for an ECG (ADInstruments, UK) placed in an Einthoven’s triangle configuration.

The ECG was sampled at 2 kHz and online filtered with the Mains Filter. RSA values were extracted for the entire duration of the baseline-block (120 s), for the last 120 s of the condition-blocks (Inclusion and Ostracism) and at Reflective stage (120 s), in line with guidelines [52].

The peak of the R-wave of the ECG was detected from each sequential heartbeat. The R-R intervals were extracted, and the artifacts were edited by integer division or summation. Editing consisted of visual detection of outlier points, typically caused by failure to detect an R-peak (e.g., edit via division) or faulty detections of two or more peaks within a period representing the R-R interval (e.g., edit via summation). The amplitude of RSA [expressed in ln (msec)^2^], calculated as the natural logarithm of the variance of heart rate activity across the frequency band associated with spontaneous respiration, was quantified with CMetX [53, 54].

### Statistical analysis

Descriptive statistics were performed to detail the socio-demographic and clinical characteristics of the sample.

### Cyberball experiment

#### Manipulation checks

Two 3*2 repeated measure analyses of variance (ANOVA) with a Group (BPD vs. rMDD vs. HC) by Condition (Inclusion vs. Ostracism) design were performed, with the post-Cyberball ratings of percentages of ball tosses received and feelings of being excluded/ignored as dependent variables.

#### Subjective responses

A 3*3 repeated-measures ANOVA was conducted with Need-Threat Scale (NTS) scores as the dependent variable to examine how the perceived threats to fundamental needs were influenced by the clinical status (Group: BPD vs. rMDD vs. HC) and by the experimental Condition (Inclusion vs. Ostracism vs. Reflective Stage).

#### Autonomic responses

Finally, a 3*4 Group (BPD vs. rMDD vs. HC) by Condition (Baseline vs. Inclusion vs. Ostracism vs. Reflective Stage) ANCOVA was used to identify whether the pattern of changes in RSA throughout the game varied among groups. Age, BMI, alcohol and tobacco consumption were considered as covariates because they could affect RSA and differed among diagnostic groups.

Simple effects analyses were used to evaluate significant main and interaction effects. All the analyses were carried out using SPSS software (IBM SPSS 25.0).

#### Association between subjective and autonomic responses to Cyberball conditions

Finally, we performed three linear regression analyses (enter method) to evaluate whether NTS scores predicted RSA following Inclusion, Ostracism and at the Reflective Stage, whilst controlling for confounding variables. In all the analyses, we entered the covariates mentioned above - age, BMI, alcohol and tobacco consumption – as well as RSA at baseline.

## Results

### Sample

The socio-demographic and clinical characteristics of the participants are shown in Table 1.

**Table 1:** Socio-demographic, clinical and psychometric characteristics

	BPD	HC	rMMD	Between-groups differences	
	n = 30	n = 30	***n*** = 30	Main effect of factor Group*	Post-hoc comparisons
**Physiological variables**					
**Age**	33.2 ± 12.07	38.9 ± 14.65	49.27 ± 9.96	F_(2,87)_ = 12.98, p < .01; ή^2^_p_ = .23	rMDD>BPD, HC (p_s_ < .01)
**Sex (F)**	27 (90%)	24 (80%)	26 (86.7%)	χ ^2^ (2) = 1.26, *p* = .53	
**Sport**	12 (40%)	17 (56.7%)	11 (36.7%)	χ ^2^ (4) = 4.57, p = .33	
**BMI**	21.77 ± 3.75	22.70 ± 3.40	26.31 ± 6.25	F_(2,87)_ = 7.98, p = .01; ή ^2^_p_ = .15	rMDD>BPD, HC (p_s_ < .01)
**Psychotropic drugs consumption**					
Alcohol	17 (56.7%)	21 (70%)	7 (23.3%)	χ ^2^ (2) = 13.87, p = .01	
Caffeine	24 (80%)	26 (86.7%)	19 (63.3%)	χ ^2^ (2) = 4.84, p = .09	
Tobacco	26 (86.7%)	8 (26.7%)	5 (16.7%)	χ ^2^ (2) = 35.02, p < .01	
**Social variables**					
**Education (yrs)**	11.53 ± 2.89	14.76 ± 3.54	11.23 ± 3.35	F_(2,87)_ = 10.77, p < .01; ή ^2^_p_ = .20	HC > BPD, rMDD (p_s_ < .01)
**Family status**					
Married/living together	7 (23.3%)	16 (53.3%)	20 (66.6%)	χ ^2^ (6) = 16.29, p = .01	
Separated/divorced	6 (20%)	1 (3.3%)	4 (13.4%)		
Widowed	0 (0%)	1 (3.3%)	1 (3.3%)		
Living alone/with parents	17 (56.7%)	12 (40%)	5 (16.7%)		
**Occupation**					
Employed	16 (53.3%)	17 (53.4%)	22 (73.3%)	χ ^2^ (6) = 29.13, p = .01	
Housewife	0 (0%)	4 (13.3%)	6 (20%)		
Students	6 (20%)	10 (33.3%)	0 (0%)		
Unemployed	8 (26.7%)	0 (0%)	2 (6.7%)		
**Clinical and psychometric variables**					
**DSM-5 Comorbidity**					
Adjustment disorder	12 (40%)	–	–		
Substance Use disorders (in full remission)	10 (33.3%)	–	–		
Alcohol Use disorders (in full remission)	3 (10%)	–	–		
Obsessive Compulsive Disorder	–	–	1 (3.3%)		
Eating disorders	2 (6.7%)	–	–		
Personality disorders	11 (36.7%)	–	3 (10%)		
Passive-aggressive	2 (6.7%)	–	–		
Paranoid	1 (3.3%)	–	–		
Histrionic	2 (6.7%)	–	–		
Narcissistic	6 (20%)	–	–		
Dependent	1 (3.3%)	–	–		
Obsessive-Compulsive	–	–	3 (10%)		
**Medications**					
Mood stabilizers	25 (83.3%)	–	4 (13.3%)	χ ^2^ (1) = 28.5, p < .01	
Antidepressants	13 (43.3%)	–	30 (100%)	χ ^2^ (1) = 24.7, p < .01	
Antipsychotics	22 (73.3%)	–	4 (13.3%)	χ ^2^ (1) = 21.08, *p* < .01	
Benzodiazepines	21 (70%)	–	14 (46.7%)	χ ^2^ (1) = 4.05, p = .04	
**Duration of illness**	15.20 ± 12.1		11.77 ± 8.77	F_(1.58)_ = 1.59, *p* = .21; ή ^2^_p_ = .03	
**Matrix reasoning**	16.80 ± 2.42	20.10 ± 2.94	18.17 ± 3.38	F_(2,87)_ = 9.52, p < .01; ή ^2^_p_ = .18	HC > BPD, rMDD (p_s_ < .04)
**GAF**	72.07 ± 7.31	96.6 ± 4.76	86.57 ± 6.39	F_(2,87)_ = 117.11, p < .01; ή ^2^_p_ = .73	HC > rMDD>BPD (p_s_ < .01)

Notes

*F-Tests in One-way ANOVA have been performed to compare continuous variables; Chi square tests (χ 2) have been performed to compare categorical variables.

BPD = patients with Borderline Personality Disorder; HC = Healthy Controls; rMDD = patients with Major Depressive Disorder in remission; BMI = Body Mass Index; GAF = Global Assessment of Functioning

The sample consisted of 90 participants, of which 85.6% were women. This gender distribution reflects the epidemiology of BPD in clinical treatment population: although in the general population there is no d ifference in the prevalence of BPD between males and females [55], in clinical settings BPD is diagnosed predominantly in females [49], likely for the greater proneness of women with BPD to seek outpatient treatment [56]. HC and patients with rMDD were matched for sex to the BPD sample, resulting in no gender differences in the three groups. In terms of age, patients with rMDD were older than both the BPD and HC groups, and, accordingly, less likely to live alone/with parents or to be students. They also had a greater BMI and reported to consume alcoholic beverages and tobacco to a lesser extent than the other groups. These differences seem to be mostly related to the age difference. As compared to the clinical groups, HC were more likely to have a college/university level of education and were all employed, with a greater level of global functioning.

### Cyberball experiment

#### Manipulation checks

As expected, there was a significant effect of the experimental *Condition* on participants’ ratings of both perception of percentages of ball throws received (*F*_1.87_ = 431.22, *p* < .01, *η*^*2*^_partial_ = .83) and feelings of being ignored and excluded (*F*_1.87_ = 184.27, *p* < .01, *η*^*2*^_partial_ = .68). As compared to the Inclusion condition, after Ostracism participants reported that they received a lower percentage of ball tosses (Ostracism 2.58 ± .37 < Inclusion 40.26 ± 1.83), and that they felt more ignored and excluded (Ostracism 4.34 ± .23 > Inclusion 1.29 ± .08). These results suggest that the *Cyberball* manipulation was successful.

Importantly, this effect held irrespective of the participants’ clinical status, as indicated by the absence of any significant *Group* effect (percentage of throws received: *F*_2.87_ = 1.91, *p* = .15, *η*^*2*^_partial_ = .04; feeling of being ignored/excluded: *F*_2.87_ = 1.13, *p* = .33, *η*^*2*^_partial_ = .03) nor *Group by Condition* interaction (percentage of throws received: *F*_2.87_ = 2.06, *p* = .13, *η*^*2*^_partial_ = .05; feeling of being ignored/excluded: *F*_2.87_ = .59, *p* = .55, *η*^*2*^_partial_ = .01. Thus, HC and participants with BPD and rMDD were equally cognitively aware of their inclusionary status during the game.

#### Subjective responses to the Cyberball game

**Fig. 2:**
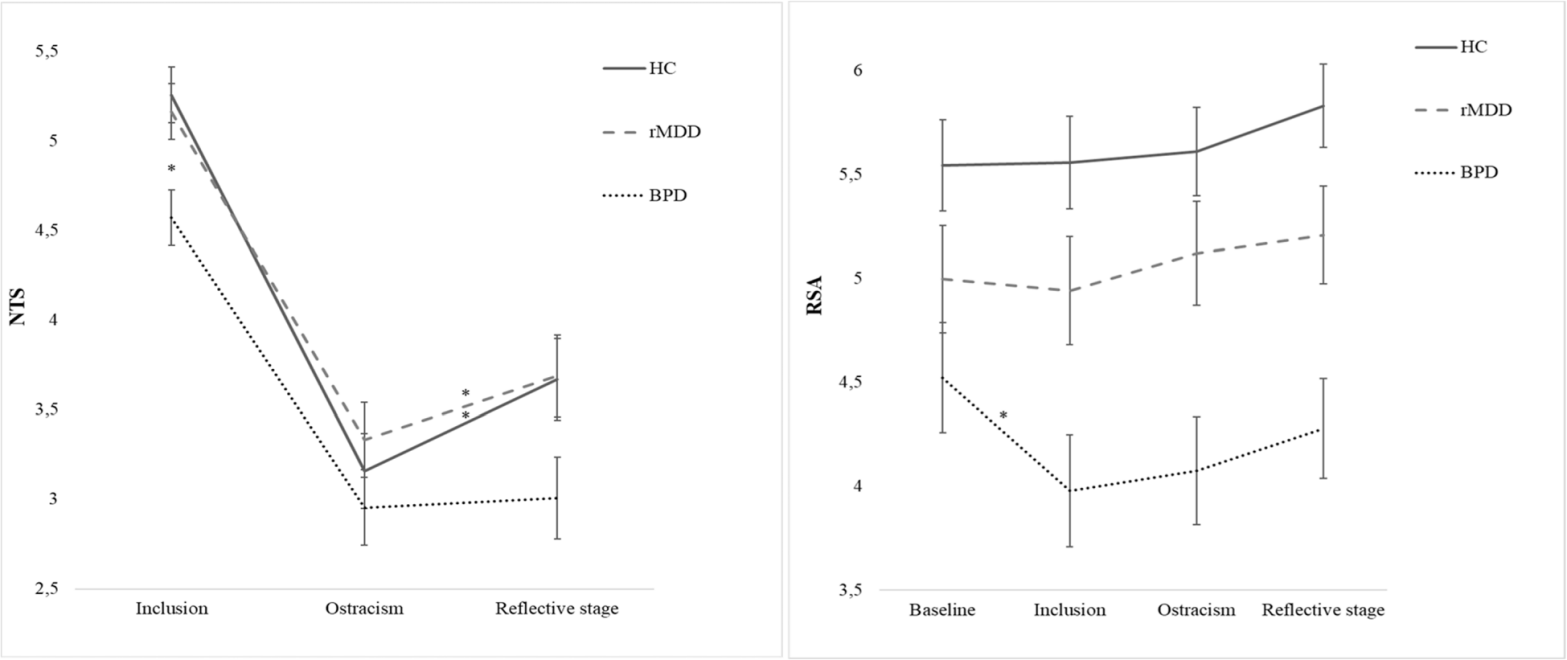
NTS scores (Panel A) and RSA levels (Panel B) across test conditions. *Note:* NTS = Need Threat Scale (higher scores represent greater satisfaction with basic needs); RSA = Respiratory Sinus Arrhythmia. Error bars depict standard error. * = *p* < .05. In *Panel A* the satisfaction with basic need (NTS, Y axis) across conditions (Inclusion, Ostracism and Reflective stage, X axis) in the three groups. After the Inclusion condition, BPD patients experienced lesser satisfaction with basic needs than HC and rMDD. Furthermore, their level of satisfaction with basic needs did not improve moving from the ostracism condition to the reflective stage. In *Panel B* Respiratory Sinus Arrhythmia (RSA, Y axis) across conditions (Baseline, Inclusion, Ostracism and Reflective stage, X axis) in the three groups. RSA decreased from Baseline to Inclusion in BPD patients, but did not vary in HC nor rMDD patients across the experimental conditions

Overall, participants reported that their fundamental needs were more threatened in the Ostracism than in the Inclusion condition (*p* < .01, CI = 1.53, 2.14) and were then restored at the Reflective stage, as compared with the Ostracism condition (*p* < .01, CI = -.51, −.11) (main within-subject effect of *Condition*; Table 2; Fig. 2A). Perception of threats to fundamental needs also varied across groups, with a tendency of BPD to report lower NTS scores (main between-subjects effect of *Group* without significant post hoc comparisons; Table 2). However, these main effects were better qualified by significant *Group by Condition* interaction (Table 2): patients with BPD reported lower satisfaction with fundamental needs than HC and patients with rMDD in the Inclusion condition (HC: *p* < .01, CI = -1.22,-.15; rMDD: *p* = .02, CI = -1.13,-.06), but not in the Ostracism condition (HC: *p* = 1, CI = -.93,.52; rMDD: *p* = .62, CI = -1.10,.35) nor at Reflective stage (HC: *p* = .13, CI = -1.26,1.45; rMDD: *p* = 1, CI = -.81,.77) (effect of *Group* within the *Group by Conditi*on Interaction for the Inclusion condition: *F*_2,87_ = 5.75, *η*^*2*^_partial_ = .12, *p* < .01). Moreover, satisfaction with fundamental needs increases from the Ostracism to the Reflective stage in HC and patients with rMDD (HC: *p* < .01, CI = -.86, −.16; rMDD: *p* = .04, CI = -.70,-.07), but not among patients with BPD (*p* = 1; CI = -.40, .30) (Effect of *Condition* within the *Group by Condition* interaction for the BPD group: *F*_2,86_ = 32.4, *η*^*2*^_partial_ = .43, *p* < .01) (Fig. 2A).

**Table 2:** Effect of Experimental Condition, Group Status and Their Interactions on NTS scores and RSA levels

Variables	BPD	HC	rMDD	Condition	Group	Interactions Condition X Group
NTS scores				F_2,86_ = 121.34^a^ η^2^_partial_ = .74 *p* < .01	F_2,87_ = 3.41 η^2^_partial_ = .07 *p* = .04	F_2,87_ = 3.24 η^2^_partial_ = .07 *p* = .04
Inclusion	4.57 ± .15	5.26 ± .15	5.16 ± .15			
Ostracism	2.95 ± .21	3.15 ± .21	3.33 ± .21			
Reflective stage	3.00 ± .23	3.66 ± .23	3.69 ± .23			
RSA levels				F_3,81_ = .29 η^2^_partial_ = .01 *p* = .84	F_2,83_ = 9.87 η^2^_partial_ = .19 *p* < .01	F_3,82_ = 3.44 η^2^_partial_ = .01 *p* = .02
Baseline	4.52 ± .27	5.54 ± .22	4.99 ± .26			
Inclusion	3.98 ± .27	5.56 ± .22	4.94 ± .26			
Ostracism	4.07 ± .26	5.61 ± .21	5.12 ± .25			
Reflective stage	4.28 ± .24	5.83 ± .20	5.21 ± .23			

BPD = patients with Borderline Personality Disorder; HC = Healthy Controls; rMDD = patients with Major Depressive Disorder in remission; NTS = Need Threat Scale; RSA = Respiratory Sinus Arrhythmia

#### Autonomic responses to the Cyberball game

Overall, patients with BPD presented a lower vagal tone as compared to HC (*p* < .01; CI = -2.22, −.63), but not to rMDD (*p* = .09; CI = -1.79, 0.85) (main between-subject effect of *Group* on RSA levels; Table 2; Fig. 2B). Specifically, BPD patients had lower resting RSA levels than HC (*p* = .02; CI = .14, 1.90), but did not differ from rMDD at baseline (*p* = .81; CI = -.57, 1.52; Effect of *Group* within a *Group by Condition* interaction for baseline RSA *F*_2.83_ = 4.43, *η*^*2*^_partial_ = .10, *p* = .02; Table 2). However, patients with BPD showed a generally lower RSA than both HC and patients with rMDD across all the experimental conditions (vs HC, all *p*_s_ < .001; vs rMDD: Inclusion *p* = .086; Ostracism *p* = .04, Reflective stage *p* = .05). Furthermore, only in the BPD group RSA levels decreased during the experiment (Effect of *Condition* within the *Group by Condition* interaction for the BPD group: *F*_3,81_ = 4.26, *η*^*2*^_partial_ = .14, *p* < .01), while they did not vary in HC nor rMDD patients (respectively, *F*_3,81_ = 1.76, *η*^*2*^_partial_ = .06, *p* = .16 and *F*_3,81_ = 1.09, *η*^*2*^_partial_ = .04, *p* = .36). In particular, patients with BPD exhibited a marked reduction in RSA from Baseline to Inclusion (*p* = .01; CI = .08, 1.01); then, their RSA levels did not vary and remained low moving from the Inclusion to the Ostracism condition (*p* = 1, CI = -.51, .31) and from Ostracism to the Reflective stage (*p* = 1, CI = -.62, .22) (Fig. 2B**)**.

#### Association between subjective and autonomic responses to Cyberball condition

Having found that participants with BPD, as compared with non-BPD controls, reported greater threats to their fundamental needs as well as a marked decrease in RSA after the *Cyberball* Inclusion condition, we next evaluated whether the subjective perception of threat in the overall sample was associated with a breakdown of vagal control at the physiological level. NTS scores predicted RSA levels only in the Inclusion condition (*b* = .22, *p* = .04), but not after Ostracism (*b* = .08, *p* = .48) nor at the Reflective stage (*b* = .04, *p* = .69). This held true even after controlling, as above, for age, BMI, alcohol and tobacco consumption and also for the baseline levels of RSA (Inclusion: *b* = .11, *p* = .05; Ostracism: *b* = −.05, *p* = .32; Reflective stage: *b* = .05, *p* = .41), indicating that higher perception of threats to fundamental needs after being included by others parallels a less efficient vagal control during including social interactions. Notably, we did not control this association for multiple comparisons.

## Discussion

The present study investigated whether BPD’s biased perception of social interactions is associated with reduced physiological regulation, as indexed by impaired vagal control, in response to experimental conditions of social Inclusion and Ostracism. Three main findings emerged.

Firstly, during the *Cyberball* task, individuals with BPD subjectively reacted to inclusion with a higher perception of threat to fundamental needs than healthy and clinical controls. Furthermore, while in non-BPD controls the fundamental needs were restored at the Reflective stage, patients with BPD did not show such recovery from ostracism. Secondly, patients with BPD presented lower resting RSA than HC, indicating stable difficulties in social predisposition. Moreover, only in patients with BPD, RSA further decreased in the inclusion condition and remained low during Ostracism and the Reflective stage. Finally, greater subjective perception of threats to fundamental needs in the Inclusion condition was associated with decreased RSA after being included, indicating that, during the *Cyberball* experiment, subjective and physiological measures of perceived threats after being included paralleled each other.

At the subjective level, after being ostracized, as expected, all participants reported being threatened in their fundamental needs [6]. However, only in the Inclusion condition patients with BPD reported a weaker sense of belongingness than did HC and patients with rMDD. This indicates that patients with BPD do not over-react to actual rejection, which is subjectively threatening for everybody; instead, they emotionally react to including interpersonal situations as they were threatening. Although patients with BPD appeared cognitively aware of the different degrees of threat conveyed by Inclusion and Ostracism and correctly estimated the percentage of ball tosses received during each condition, they subjectively perceived a higher level of danger in the including and accepting interaction than non-BPD participants. This is in line with previous *Cyberball* studies showing that patients with BPD during the Inclusion condition experienced a greater sense of exclusion and a lesser sense of inclusion and belonging [8–11, 57] and reported lower feelings of social connection and greater threats to their social needs than controls even when over-included by others [11, 12]. Thus, patients with BPD show a biased subjective experience of social inclusion during *Cyberball* [8, 57].

Furthermore, in line with the Temporal Need-Threat Model of ostracism [51], in this study, threatened needs quickly recovered a few minutes after Ostracism among both HC and rMDD. While the detection of ostracism immediately generates negative emotions, this in turn quickly motivates individuals to regulate their initial social pain in order to access more positive emotions and restore functional relations with others after Ostracism is over. Conversely, in this study patients with BPD did not recover from Ostracism; rather, they kept reporting feeling threatened in their need to belong. The ability to recover faster and in more functional ways from social exclusion has been found in individuals with higher psychological flexibility levels. By contrast, a delayed emotional recovery suggests difficulties in access to, and use of, a wider range of emotion regulation strategies to cope with ostracism experiences [58, 59]. For instance, socially anxious individuals exhibit a slow recovery from the negative feelings induced by ostracism [60].

This study also demonstrated that these explicit, subjective findings parallel a corresponding pattern of change in ANS reactivity at the implicit, physiological level. BPD patients exhibited a lower RSA at baseline than HC. Moreover, they also uniquely showed a further decrease in RSA after the *Cyberball* Inclusion condition – when they also reported, at the subjective level, to be threatened (more than non-BPD controls) in their need to belong.

Concerning baseline RSA, the present findings confirm that patients with BPD show lower vagal control at rest than HC [17–21]. This indicates that even in the absence of interpersonal challenges (i.e., even before the *Cyberball* experiment), the BPD group exhibits a physiological state of preparedness for defensive rather than prosocial behaviors. Notably, the finding of low RSA at rest in clinical populations, as compared to HC, is not limited to BPD but also characterizes patients with anxiety disorders, conduct disorders, autism spectrum disorders, depression and schizophrenia (see for a review [61]) and, in this study, also patients with MDD in remission. This is not surprising given that patients with diverse psychiatric disorders exhibit various degrees of social dysfunction that are likely to be paralleled, at the ANS level, by the inhibition of the social engagement system when the vagal brake is removed. This study extends these findings by indicating that even after clinical remission patients with MDD maintain a state of physiological arousal predisposing to defensive rather than prosocial behaviors; consistently, they also showed lower psychosocial functioning than HC, as indexed by lower GAF scores, possibly as a “scar effect” of previous episodes. Thus, low RSA at rest, rather than being disorder-specific, could represent a marker of the difficulties in social behaviors shared by several psychiatric disorders.

However, among HC and patients with rMDD, RSA levels did not change from baseline across the three *Cyberball* experimental conditions. This indicates that they correctly appraised, at the physiological level, that the task conveyed only minimal interpersonal stress (*Cyberball* lasted about two minutes and involved two unknown avatars online), but not greater threatening risks. Thus, the experimental *Cyberball* conditions employed in this study ultimately favored, among HC and patients with rMDD, the maintenance of their vagal regulation on the heart during and after the experiment. Therefore, while at the subjective level both HC and patients with rMDD accurately perceived the Ostracism condition as threatening their need to belong, they maintained their capacity to regulate the vagal control and then quickly recovered from their negative affective states at the Reflective stage. On the contrary, patients with BPD experienced a further drop in RSA after the benign experimental condition of Inclusion. This vagal withdrawal then persisted even at the reflective stage. Thus, patients with BPD are not only biased to subjectively perceive rejection even in including contexts, or when ostracism is over: they also implicitly appraise, at the physiological level, such benign conditions as signaling threats in the environment. This altered neuroception [13] of favorable social environments, in turn, leads BPD patients to regulate their ANS to a state that would support fight and flight responses but impedes social flexibility and prosociality even after including interpersonal exchanges or when social interactions are over. These findings are in keeping with other studies investigating the potential neurophysiological bases of BPD biased perception of rejection. For instance, during the *Cyberball* Inclusion condition, patients with BPD show an enhanced P3b event-related potential, which usually signals social rejection [12, 62] and hyper-activate the “social pain” neural circuitry (i.e., the dorsal anterior cingulate cortex and the dorsomedial prefrontal cortex) [10]. These data suggest that patients with BPD process objectively positive social interactions by activating physiological and neural responses that signal rejection and threat.

Finally, in this study, the subjective appraisal of threats to one’s need to belong after the Inclusion condition (NTS scores) directly correlated with the physiological appraisal of the Inclusion condition as unsafe (decreased RSA ratings after Inclusion), but not in any other stage of the *Cyberball* task. This indicates that the tendency to subjectively perceive including interpersonal interactions *as if* they were excluding is underlined by a corresponding altered physiological appraisal of such safe context *as if* it was unsafe and risky. Such altered appraisal inevitably leads to the inappropriate activation of the ANS defensive systems in an environment that is actually safe and inhibits the prosocial responses fostered by the myelinated vagal regulation, which though would be required and adaptive in safe contexts [13].

Overall, these results point out to a “lowered ostracism detection threshold” in BPD: when the threshold for detecting signals of ostracism in the environment is set too low, the ostracism detection system registers a high proportion of false positives, interpreting benign (or even mildly favorable) interpersonal events as potential threats to acceptance [63]. Such interpretation is supported by converging lines of evidence indicating that patients with BPD systematically underestimate positive feedback from others. For instance, they show lesser expectations of being socially accepted than controls and cannot adjust these expectations even after receiving actual positive feedback [64]. Furthermore, in behavioural economics games, BPD under-notice others’ fair behaviour toward them and react to that as if it was unfair by punishing them [65]. Moreover, after experiencing actual social acceptance, they behave less cooperatively toward others [64]. Finally, individuals with BPD respond with less positive emotions than controls to others’ friendly behaviour [66], and under-notice trust in others [67–69].

These findings are also consistent with clinical observations that patients with BPD do not seem to benefit from benign, “fair” and accepting attitudes of others toward them to regulate their emotional states, nor from neutral interpersonal conditions where interpersonal rejection, although experienced in the past, is no longer occurring. According to object-relations theory [70, 71], this response pattern may reflect the patient’s unconscious idealized need of finding a perfectly “accepting” relationship with others. However, this intense need is unlikely to be fulfilled in reality, since human interactions may also exhibit transient difficulties or ruptures that are usually overcome by repairing trust and maintaining reciprocity. For patients with BPD, though, such less-than-perfect interpersonal interactions may not be enough to fulfill their unconscious idealized need for interpersonal belonging. Thus, in the desperate attempt to protect this unconscious hope of a “perfect” relationship, individuals with BPD need to project one’s negative affect into the others; this makes them perceive including social interactions as if they were unfair and excluding. This threatens the possibility to feel safe and connected during “real” interpersonal exchanges.

Such dynamics could have significant implications for treatment. Patients with BPD could feel easily threatened, hurt and ignored even in the context of therapeutic relationships, and may find it difficult to appraise them as trustworthy, regardless of the objectively cooperative stance of the therapist. This may affect the therapeutic alliance and possibly evoke negative countertransference reactions in the clinicians, such as feelings of frustration, inadequacy, and hopelessness. It is hence important, for the clinician, to recognize that these feelings in fact correspond to some aspects of their patients’ inner experience that they cannot tolerate. By maintaining this empathic focus, the therapist can then explore with the patients their perception to be threatened during treatment and help them to appreciate that, although the therapeutic relationship cannot provide a perfect or magical solution to their problems, it can nonetheless represent something of value to them. In the same vein, the clinicians may encourage BPD patients to recognize positive aspects in their real-life interpersonal relationships by clarifying the defensive distortion of benign interpersonal encounters as threatening. In turn, this will favor the development of more gratifying and satisfactory interpersonal relationships [72].

The results of this study should be interpreted in light of some limitations.

First, our patients with BPD and rMDD exhibited some socio-demographical differences, above all age. This reflects the epidemiological distribution of BPD and MDD. While BPD has an onset in adolescence or early adulthood, and most patients experience symptomatic remission in a few decades [73], MDD can develop at any age, with a median age at onset at 30–40 years [74].

Moreover, we could not rule out the role of pharmacotherapy on RSA levels, since both the clinical groups kept their usual medication regimen, in compliance with the Local Ethical Authority requirements and good clinical practice. Nonetheless, in this study, patients with (*N* = 43) and without (*N* = 17) antidepressants did not differ in baseline RSA (*F*_1.51_ = .38, *p* = .54, *η2*_partial_ = .01), regardless of their clinical group status. Moreover, in our analyses, we controlled for other physiological variables that have previously been demonstrated to affect RSA (i.e., age, BMI, alcohol and tobacco consumption) [75–78].

Furthermore, while in the present study healthy and clinical controls subjectively perceived the ostracism condition as potentially threatening (thereby confirming the widely-replicated validity of the *Cyberball* experimental manipulation), at the physiological level they did not exhibit a parallel withdrawal in RSA. This is likely due to the successful recruitment of self-regulatory abilities in HC and in patients with rMDD, which favored the appraisal, at the physiological level, of the *Cyberball* ostracism condition as a minimal and transient interpersonal stress, and therefore the maintenance of vagal control. This was not the case for patients with BPD, who are known to react with increasing distress to any situation where rejection is a possibility (in the present study, to the *Cyberball* social inclusion condition) because of their self-regulation difficulties [79, 80]. A subsequent vagal suppression did not occur from inclusion to ostracism, among patients with BPD, because it was already withdrawn from baseline to inclusion, and RSA reactivity scores could be susceptible to a floor effect of functional adaptations [81].

Finally, in our study, we interpreted RSA in the Polyvagal Theory framework, which posits that RSA suppression is associated with the neurophysiological appraisal of the environment as dangerous, thus leading to defensive, rather than pro-social behaviors. However, contradictory findings have been reported regarding RSA changes in response to varying environmental cues [82] and some researchers suggested that the evolution of the parasympathetic ANS has a greater anatomical and physiological complexity than what was proposed by the Polyvagal Theory [16, 83]. Therefore, the specificity of RSA as a physiological marker of BPD patients’ biased perception of social participation needs to be confirmed by further research.

## Conclusions

The results of this study indicate that patients with BPD perceive (at the subjective level) threats to their need to belong during accepting social encounters, as well as when the experience of ostracism is no longer present. They also appraise (at the implicit, physiological level) such circumstances as threatening and dangerous, thereby showing an autonomic response characterized by increased physiological arousal and proneness to defensive reactions and breakdown in prosocial behavior. These findings support the view that patients with BPD appraise and react, both subjectively and physiologically, to positive social contexts as if they were unsafe and rejecting. This prevents them from appreciating and reciprocating objectively inclusive, “fair” social exchanges. Thus, individuals with BPD may benefit from interventions that help them to accurately appraise positive cues in their social and interpersonal interactions.

## Data Availability

The dataset analyzed during the current study is available from the corresponding author on reasonable request.

## Abbreviations

ANOVA: analyses of variance
ANS: Autonomic Nervous System
BMI: Body Mass Index
BPD: Borderline Personality Disorder
ECG: Electrocardiogram
GAF: Global Assessment of Functioning
HAM-A: Hamilton Anxiety Rating Scale
HAM-D: Hamilton Rating Scale for Depression
HC: Healthy Controls
NTS: Need-Threat Scale
rMDD: remitted Major Depressive Disorder
RSA: Respiratory Sinus Arrhythmia
SCID-5-PD: Structured Clinical Interview for DSM-5 Personality Disorders
SCID5-CV: Structured Clinical Interview for DSM-5 disorders, Clinician Version
