# Correction to: Autonomic vulnerability to biased perception of social inclusion in borderline personality disorder

**DOI:** 10.1186/s40479-021-00170-w

**Published:** 2021-12-09

**Authors:** Maria Lidia Gerra, Martina Ardizzi, Silvia Martorana, Veronica Leoni, Paolo Riva, Emanuele Preti, Barbara Francesca Marta Marino, Paolo Ossola, Carlo Marchesi, Vittorio Gallese, Chiara De Panfilis

**Affiliations:** 1 Department of Mental Health, AUSL of Parma, Parma, Italy; 2 Department of Medicine and Surgery, University of Parma, Parma, Italy; 3 Department of Psychology, University of Milano-Bicocca, Milan, Italy

**Correction to: bord personal disord emot dysregul 8, 28 (2021).**

**https://doi.org/10.1186/s40479-021-00169-3**

Following publication of this article [1], it is reported that the given names and family names of the authors were interchanged. The correct author names are displayed below.

Maria Lidia Gerra.

Martina Ardizzi.

Silvia Martorana.

Veronica Leoni.

Paolo Riva.

Emanuele Preti.

Barbara Francesca Marta Marino.

Paolo Ossola.

Carlo Marchesi.

Vittorio Gallese.

Chiara De Panfilis.

The original article has been updated.

## References

[1] Gerra ML, Ardizzi M, Martorana S, et al. Autonomic vulnerability to biased perception of social inclusion in borderline personality disorder. bord personal disord emot dysregul. 2021;8(28):28. 10.1186/s40479-021-00169-3. 10.1186/s40479-021-00169-3 PMC8600701 34794518

